# Cardiovascular disease risk factors among undergraduate medical students in a tertiary care centre of eastern India: a pilot study

**DOI:** 10.1186/s43044-021-00219-9

**Published:** 2021-10-26

**Authors:** Somnath Mukhopadhay, Anindya Mukherjee, Dibbendhu Khanra, Biaus Samanta, Avik Karak, Santanu Guha

**Affiliations:** 1 Asansol District Hospital, Asansol, India; 2 Department of Cardiology, NRS Medical College, 138, A.J.C. Bose Road, Kolkata, 700014 India; 3 Royal Wolverhampton NHS Trust, Wolverhampton, UK; 4 Department of Cardiology, Medical College, Kolkata, India; 5 Department of Cardiology, AMRI Hospitals, Kolkata, India; 6 Department of Cardiology, Ruby General Hospital, Kolkata, India

**Keywords:** Cardiovascular disease risk, MBBS students, Waist-to-height ratio, Young population

## Abstract

**Background:**

Handful studies report the prevalence of cardiovascular disease (CVD) risk factors among medical students from India and none from the eastern part of the country.

**Aim:**

To estimate the prevalence of risk factors of CVD and their correlation with CVD risk ratio among the MBBS students from eastern India.

**Methods:**

433 students were studied. International Physical Activity Questionnaire-long form was used for assessment of physical activity and Perceived Stress Scale (PSS) to elicit psychological stress levels. Waist-to-height ratio (WHtR) was calculated. Total cholesterol to high-density lipoprotein ratio was calculated as the CVD risk ratio.

**Results:**

39.3% were women and 68.6% of the subjects were in junior classes. 22.4% subjects had high PSS while 30% performed low physical activity. Tobacco and alcohol intake was prevalent in 29.3% and 21.0% respectively. High CVD risk ratio was found in 14.3%. Most risk factors were more prevalent among juniors except diabetes. Among the non-overweight and non-obese subjects there was a significant positive correlation between WHtR and CVD risk score (*R* = 0.33, *p* < 0.001). 82.7% of the variance in CVD risk ratio could be explained by WHtR, Body mass index, Triglycerides and Low-density lipoprotein (*F*(7, 425) = 296.085), of which LDL (*β* = 0.755) contributed the most.

**Conclusions:**

High prevalence of different modifiable CVD risk factors revealed among the subjects in this study is concerning. WHtR appears promising as an independent early predictor of CVD risk in Indian population. A dedicated CVD risk assessment tool for the young population is necessary.

**Supplementary Information:**

The online version contains supplementary material available at 10.1186/s43044-021-00219-9.

## Background

Cardiovascular diseases (CVDs) lead the causes of morbidity and mortality worldwide and more than 75% of Cardiovascular diseases related deaths occurs in low- and middle-income countries [1, 2]. It is the leading cause of death in India and Cardiovascular diseases deaths occur a decade earlier in Indians than Western countries [3, 4].

Atherogenic diet, sedentary lifestyle and ‘South Asian lipid profile’ are the most important behavioural risk factors for insulin resistance which leads to metabolic syndrome, diabetes mellitus, obesity and eventually Cardiovascular diseases [5].

Long term Cardiovascular diseases risk in young adults can be strongly predicted by their risk factor profiles [6]. The magnitude and types of risk factors among young adults needs to be understood to establish targeted intervention through lifestyle changes [6, 7]. Most of the young adults are not aware of the Cardiovascular diseases risk and do not undergo screening leading to risk underestimation in spite of high prevalence [6]. Hence, the urgent need of addressing the prevalence of Cardiovascular diseases risk factors among young adults.

Undergraduate medical students form a very important subset of young adults in any society since they are the forming elements of the future healthcare infrastructure. The stress associated with intense training in medical school and lack of physical exercise puts the students at higher risk of Cardiovascular diseases [8]. Various studies have shown the high prevalence of Cardiovascular diseases risk factors among medical or nursing students [8–12]. The awareness levels of medical students regarding Cardiovascular diseases risk factors needs further insight and improvement [13, 14]. In India there has been only handful studies reporting the prevalence of Cardiovascular diseases risk factors among medical students and none from the eastern part of the country [8–10]. Prevalence of Cardiovascular diseases risk factors vary across ethnicity and races [14].

The objective of the current study was to estimate the prevalence of risk factors of Cardiovascular diseases and their association with Cardiovascular diseases risk ratio among the undergraduate medical students of a tertiary care facility of eastern India.

## Methods

### Subjects/study design

The study employed the descriptive, cross-sectional approach. Undergraduate medical students pursuing Bachelor of Medicine, Bachelor of Surgery (MBBS) course, who did not have known cardiovascular diseases, were recruited from 1 May 2019 to 31 July 2020. The students in 1st and 2nd year of their MBBS curriculum have been referred to as the junior students and those in 3rd and final years as senior students in the following text. Purposive sample size was calculated to be ≥ 385 to have confidence level of 95% so that the real values were within ± 5% of measured or surveyed value [15]. All procedures followed were in accordance with the ethical standards of the responsible committee on human experimentation (institutional and national) and with the Helsinki Declaration of 1964 and later revisions. Informed written consent was obtained from all subjects for being included in the study.

### Questionnaire

An anonymous, confidential, self-administered structured questionnaire was used which included their age, gender, year of education, family history of Cardiovascular diseases, tobacco intake and alcohol use. Tobacco intake or alcohol use was defined as history of regular or occasional tobacco or alcohol use respectively [16]. Urban and rural residence was defined as per the Indian census guidelines [17]. Family history of Cardiovascular diseases was defined as a self-reported diagnosis of Cardiovascular diseases in parents, siblings, or children that occurred at 60 years or younger [18]. Apart from these, 2 pre-validated questionnaires were included in the structured questionnaire- International Physical Activity Questionnaire (IPAQ)-Long form for assessment of physical activity and Perceived Stress Scale (PSS) to elicit psychological stress levels [19, 20]. Physical activity was classified as low, moderate and high as per International Physical Activity Questionnaire (IPAQ)-Long form. Scores ranging from 0 to 13 in physical activity and Perceived Stress Scale (PSS) were considered low stress, 14–26 were considered moderate stress and 27–40 were considered high perceived stress.

### Anthropometric measurements

Body weights were measured with the HBF-516 Body Composition Monitor and Scale (IL, USA). Waist circumference was measured at the midpoint between last rib and iliac crest [21]. SecaStadiometer (Hamburg, Germany) was used to measure the heights to the nearest 0.1 cm. Waist-to-height ratio (WHtR) was calculated and a boundary value of 0.5 was used [22]. Body mass index was calculated from height and weight with following categorization: Underweight (< 18.0 kg/m^2^), normal body mass index (18.0–22.9 kg/m^2^), overweight (23.0–24.9 kg/m^2^), obese (> 25 kg/m^2^) [23].

### Blood pressure, glucose and lipid profile

The subjects sat in a relaxed and comfortable position and all measurements were taken on the right arm with Omron digital blood pressure monitor (IL, USA). 2018 European Society of Cardiology (ESC) hypertension guidelines were followed in measuring and categorizing blood pressure thus defining hypertension as systolic blood pressure (SBP) ≥ 140 mm Hg and/or diastolic blood pressure (DBP) ≥ 90 mm Hg [24].

3 mL of venous blood was collected into gel separator tubes for lipid profiling after 12 h of fasting. After centrifugation at 3000 rotation per minute for 10 min, the serum samples were used for analysis by Vitros 5-IFS chemistry analyzer (NY, USA). The amount of low-density lipoprotein cholesterol (LDL) in each serum was obtained using the Friedewald’s equation: low-density lipoprotein cholesterol (LDL) = Total cholesterol (TC)—high-density lipoprotein cholesterol (HDL-C)—(Triglyceride [TG]/2.2) [25]. The ranges (in mg/dL) were categorized as follows: Triglyceride < 150- acceptable, ≥ 150- high; Total cholesterol < 200- acceptable, ≥ 200- high; low-density lipoprotein cholesterol ≤ 100- acceptable, > 100- high; and high-density lipoprotein cholesterol ≤ 40 (men)- low, > 40 (men)- acceptable, ≤ 50 (women)- low, > 50 (women)- acceptable [21]. Total cholesterol to high-density lipoprotein cholesterol ratio was calculated as the cardiovascular diseases risk ratio and categorized as ≤ 4.5 (men)-acceptable, > 4.5 (men)- high, ≤ 4.0 (women)-acceptable, > 4.0 (women)-high [26].

Diabetes was defined as fasting plasma glucose (FPG) ≥ 126 mg/dL where fasting was defined as no caloric intake for at least 8 h. In the absence of unequivocal hyperglycemia, diagnosis was done based on two abnormal test results from the same sample or two separate test samples [27].

### Data analysis

Statistical package for Social Science (SPSS) version 25 (SPSS Inc, Chicago, Ill., USA) was used to analyse the data. Mean and standard deviation (SD) for continuous variables and percentages for categorical variables were calculated as part of descriptive analysis. Continuous variables were compared by Student’s t test while categorical variables were compared by Chi-square test. A p value of less than 0.05 was considered statistically significant. Associations were assessed using Chi-Square tests of association. Correlation between the cardiovascular risk factors was assessed by Pearson’s correlations. Multiple linear regressions were used to assess the extent of independent contribution of variables to the development of Cardiovascular diseases risk.

## Results

### Baseline characteristics

433 undergraduate medical students were included of whom 263 were men and 170 were women. 68.6% of the subjects were in junior classes. 62.1% students belonged to urban population. Mean Triglyceride, Total cholesterol, low-density lipoprotein, waist circumference, diastolic blood pressure and cardiovascular risk ratio were significantly higher in men; mean high density lipoprotein was significantly lower in men. Baseline characteristics have been outlined in Table 1. 21.2% were hypertensive and 13.4% were diabetic. None of the students were married or on oral contraceptive pills.

**Table 1:** Background characteristics of students

Variable	Male (*n* = 263)	Female (*n* = 170)	Total (*n* = 433)	Significance
Age (years)	22.5 ± 1.9	21.8 ± 2.2	22.2 ± 2.1	0.001*
Junior	197 (74.9)	100 (58.8)	297 (68.6)	0.001*
Senior	66 (25.1)	70 (41.2)	136 (31.4)	
Urban	152 (57.8)	117 (68.8)	269 (62.1)	0.02*
Rural	111 (42.2)	53 (31.2)	164 (37.9)	
BMI (kg/m^2^)	25.5 ± 3.9	25.5 ± 3.7	25.5 ± 3.9	0.9
TG (mg/dL)	147.7 ± 74.6	125.8 ± 59.0	139.1 ± 36.7	0.001*
TC (mg/dL)	174.0 ± 39.5	162.2 ± 30.6	169.3 ± 36.7	0.001*
HDL (mg/dL)	51.7 ± 9.9	54.8 ± 9.4	52.9 ± 9.8	0.001*
LDL (mg/dL)	92.8 ± 34.7	82.2 ± 26.1	88.6 ± 32.0	0.001*
FPG (mg/dL)	98.0 ± 23.8	97.4 ± 20.7	97.8 ± 22.6	0.8
WHtR	0.48 ± 0.05	0.49 ± 0.05	0.49 ± 0.05	0.02*
SBP (mmHg)	127.3 ± 12.6	126.0 ± 12.5	126.8 ± 12.6	0.3
DBP (mmHg)	82.2 ± 7.3	78.6 ± 6.7	80.8 ± 7.3	< 0.001*
Cardiovascular risk ratio	3.5 ± 1.1	3.1 ± 0.8	3.3 ± 1.0	< 0.001*

BMI, body mass index; DBP, diastolic blood pressure; FPG, fasting plasma glucose; HDL, high density lipoprotein; LDL, low density lipoprotein; SBP, systolic blood pressure; TC, total cholesterol; TG, triglyceride

*Test significant at *p* < 0.05. Continuous variables are expressed as mean ± SD (corrected up to 1st decimal) and discrete variables are expressed as *n* (%)

### Cardiovascular diseases risk prevalence

#### Gender wise prevalence

**Fig. 1:**
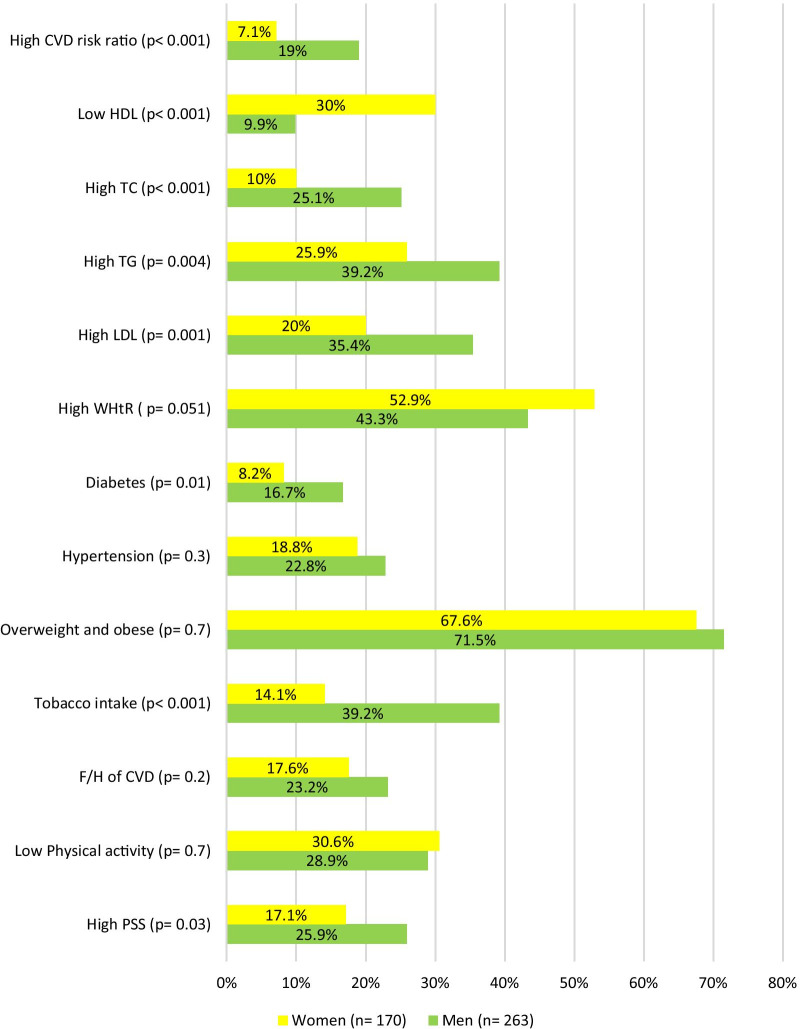
CVD risk factor prevalence among male and female students

Significantly higher proportion of men (25.9%) had high perceived Stress Scale (PSS) than women (17.1%) (*p* 0.03). Approximately 30% of both men and women had low physical activity while none in the study reported of high physical activity. Tobacco and alcohol intake was significantly higher in men (39.2% and 31.6% respectively) than women (*p* < 0.001). High low-density lipoprotein (35.4%), triglyceride (39.2%) and total cholesterol (25.1%) were significantly more frequent in men while low high-density lipoprotein was significantly more frequent in women (30%) (*p* < 0.005). Diabetes was more prevalent among men (*p* = 0.01). High cardiovascular disease risk ratio was found to be significantly more common in men (19%) (*p* < 0.001). The gender wise prevalence is outlined in Fig. 1 and detailed gender wise distribution is tabulated in Additional file 1: Table S1.

#### Seniority wise prevalence

**Fig. 2:**
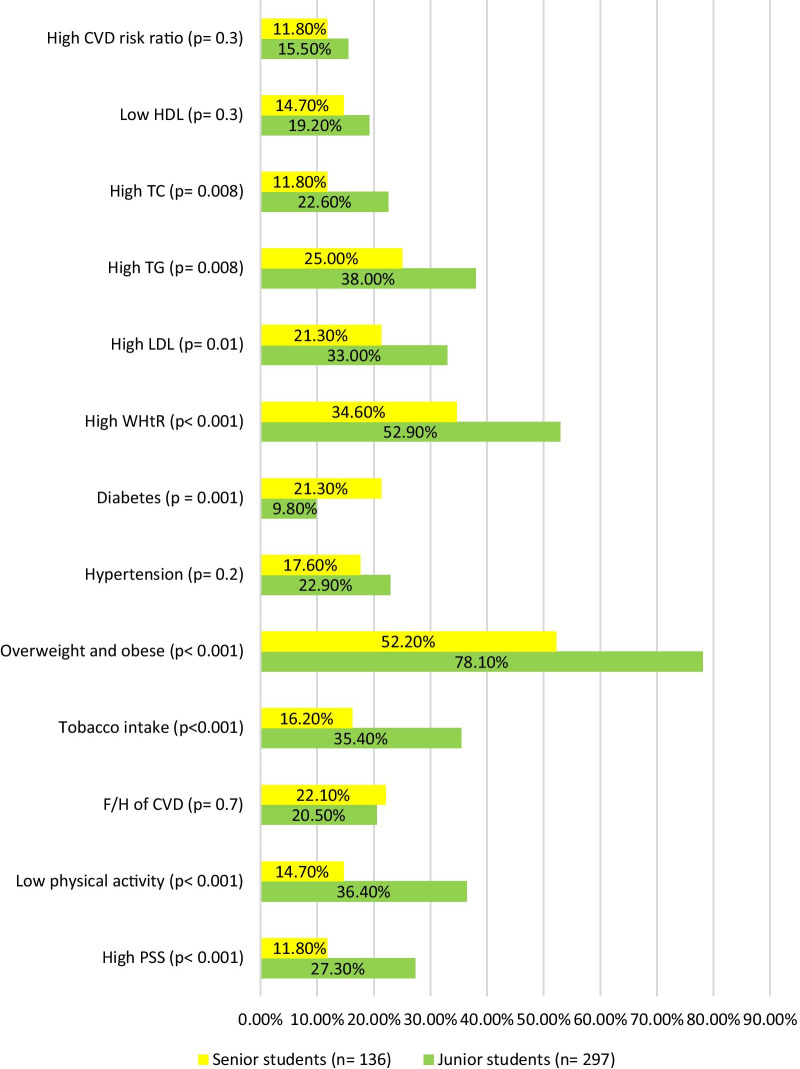
Cardiovascular risk factor prevalence among junior students and senior students

High Perceived Stress Scale (27.3%), low physical activity (36.4%), F/H of diabetes mellitus (DM) (33.7%), tobacco intake (35.4%), alcohol intake (24.9%), overweight (20.9%), obesity (57.2%) was significantly more common among juniors (*p* < 0.05). Diabetes was more prevalent among seniors (*p* = 0.001). Similarly, increased waist-height ratio (WHtR) (52.9%), high low-density lipoprotein (33%), high triglyceride (38%), high total cholesterol (22.6%) were significantly more common among junior students (*p* < 0.05). The seniority wise prevalence is outlined in Fig. 2 and detailed seniority wise distribution is tabulated in Additional file 1: Table S2.

#### Association and correlation

**Fig. 3:**
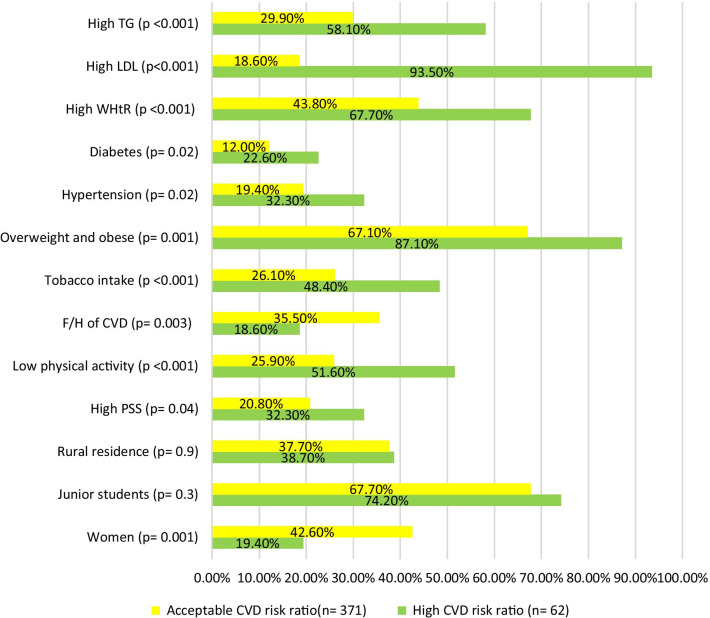
Association of the baseline parameters and CVD risk factors with CVD risk ratio

Male gender, high PSS, low physical activity, tobacco intake, overweight and obesity, hypertension, diabetes, high waist-height ratio (WHtR), high triglyceride and high low-density lipoprotein had significant association with high cardiovascular disease risk ratio as outlined in Fig. 3. Additional file 1: Table S3 details the statistics of this analysis.

**Fig. 4:**
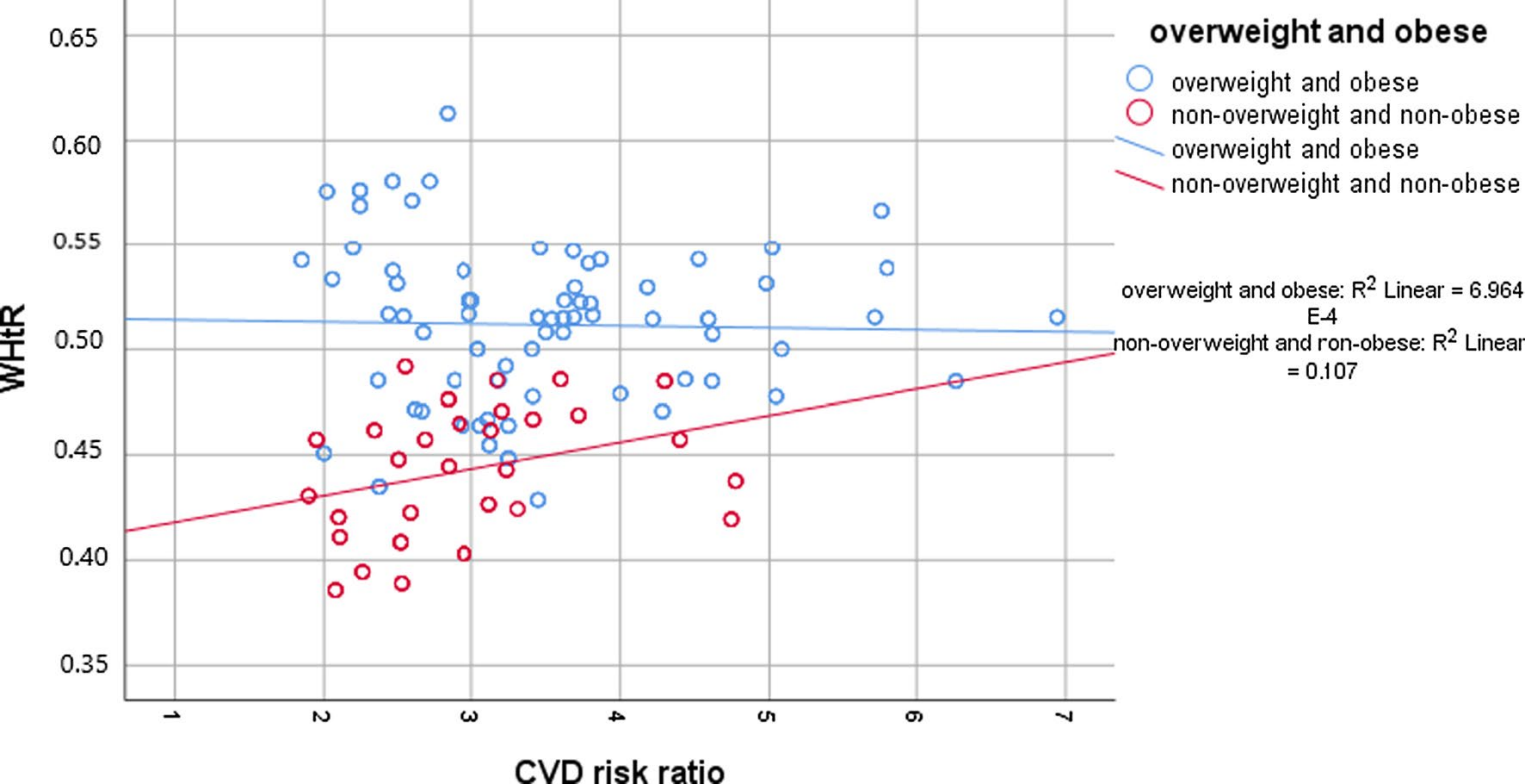
Correlation between WHtR and CVD risk ratio stratified by overweight and obesity

Waist-height ratio and cardiovascular risk score was weakly correlated with Pearsons’s *R* = 0.19 (*p* < 0.001) across overall population. Among overweight and obese subjects there was no significant correlation (*p* = 0.6) while among the non-overweight and non-obese subjects there was a weak but significant positive correlation (*R* = 0.33, *p* < 0.001) as depicted in Fig. 4.

## Discussion

The INTERHEART and INTERSTROKE study defined the impact of the 9 potentially modifiable risk factors (diabetes, hypertension, smoking, lipids, obesity, diet, physical activity, alcohol consumption and psychosocial factors) on cardiovascular disease and thus the approach to prevention of the same [28, 29]. Our study aimed to assess the cardiovascular disease risk factors among the undergraduate medical students from a tertiary centre of eastern India. Being the first study from this part of the country, this sheds light on the risk factors among medical students that needs to be intervened upon to improve the quality of life of future doctors. Comparison of the data with other recent studies conducted on medical students in different parts India have been discussed in Table 2.

**Table 2:** Prevalence of CVD risk factors among medical students in different studies from India

Study	Mukhopadhay et al. (2021)	Rustagi et al. [30]	Paul B et al. [9]	Kurian et al. [31]	Nanjesh et al. [10]	Ameer Khan et al. [8]
Sample size	433	433	458	144	500	396
Grades	Undergraduate	Undergraduate	Undergraduate	Undergraduate	Undergraduate	–
Site	East India	North India	South India	South India	South India	South India
F/H of CVD (%)	21.0	–	64.2	12.0	14.2	8.1
Tobacco intake (%)	29.3	7.0	5.0	0.0	11.6	4.0
Alcohol intake (%)	21.0	28.8	5.9	0.0	27.0	–
Low physical activity (%)	29.6	42.6	47.6	13.0	18.0	13.6
Overweight (%)	16.4	–	38.2^b^	15.0	29.0	–
Obese (%)	53.6	–		12.0	5.8	
High SBP (%)	18.5	–	27.1^a^	10.0	1.2^a^	2.0^a^
High DBP (%)	13.9	–		21.0		
Increased waist circumference (%)	26.1	–	–	23.0	15.2	–
High LDL (%)	29.3	–	–	5.0	–	–
High TG (%)	33.9	–	–	13.0	–	–
High TC (%)	19.2	–	–	8.0	–	–
Low HDL (%)	17.8	–	–	33.0	–	–
High CVD risk ratio (%)	14.3	–	–	16.0	–	–

DBP, diastolic blood pressure; CVD, cardiovascular disease; HDL, high density lipoprotein; LDL, low density lipoprotein; SBP, systolic blood pressure; TC, total cholesterol; TG, triglyceride

^a^Included all high BP

^b^Included overweight and obese

### Stress

High perceived Stress Scale was noted in 22.4% of the subjects in this study. The study by Khan AAA et al. found stress to be present in 25.5% medical students and sadness or depression in 3.0%. Stress was detected in 22.5% of the Indian urban affluent adults aged 30–45 years in the study by Aeri et al. [32]. The medical students were having similar stress levels at a lesser age which is concerning. Similarly, a Portuguese study found extremely high levels of stress in 20.8% of sixth year medical students and a study from Kingdom of Saudi Arabia reported severe stress in 21.8% of the female medical students [33, 34]. High perceived Stress Scale was noted in significantly more proportion of junior students which can be due to the more sedentary lifestyle, computer-based classes and being away from home in the pandemic and the stress of the medical curriculum which the senior students get used to with passing years of education.

### Physical activity

29.6% of the students in this study had low physical activity. Physical activity was nil or occasional among 42.6% subjects in the study by Rustagi et al. [33]. Paul B et al. also reported sedentary activity more than 4 h per day in 47.6% of the students [9]. Paul B also found men to be more active than women but in our study there was no significant gender based difference [9]. In contrary, number of subjects performing low total physical activity was lower in the study by Kurian S et al. (13.0%), Nanjesh SK et al. (18.0) and Thomas A et al. (13.6%) [8, 10, 31]. Low physical activity has been reported among university students (22–62%) from other studies as well [30]. Ibrahim et al. reported physical inactivity in 57.9% of the medical students in King Abdulaziz University, Jeddah, Saudi Arabia whereas the study by Boo NY from Malaysia reported physical inactivity in 29.9% of the medical students which was similar to our study population [11, 35]. This can be due to the busy academic schedules of students and overall inclination towards sedentary habits and technology-based lifestyle. In this study junior students were more inclined towards lower physical activity probably because of their computer-based classes in the covid pandemic and lack of clinical ward round based classes in junior years.

### Family history

Family history of cardiovascular disease was present in 21% of the subjects. Family history of dyslipidemia and diabetes was also prevalent in 21.5% and 30.0% students respectively, these nearly similar proportions reflecting the fact that dyslipdemia, diabetes and cardiovascular disease are closely related and goes hand in hand. Family history of cardiovascular disease varied widely across the different studies done across the Indian subcontinent from 64.2% in the study by Paul B et al. to 8.1% in the study by Thomas A et al., 14.2% in the study by Nanjesh SK et al. and 12.0% in that by Kurian et al. [8–10, 31]. 56.8% of medical students from a medical college in Karachi were reported to have family history of cardiovascular disease whereas the prevalence of family history of premature coronary artery disease was 14.4% in Saudi Arabian medical students and 6.7% in Egyptian medical students in another study [36, 37]. This variation can be due to socio-demographic differences across the populations.

### Tobacco and alcohol intake

29.3% of the students were tobacco users which was significantly higher than the values reported in other Indian studies but similar to the data from 2 studies among Egyptian and Saudi Arabian students where smoking was practiced by 29.7%.and Croatian medical students where 30.4% were smokers [8–10, 13, 30, 37]. Smoking was noted in 4.9% of medical students from International Medical University, Malaysia and 2.8% among students of King Abdulaziz University, Jeddah, Saudi Arabia [11, 35]. This variation can be due to absence of strict non-smoking laws or awareness programs in the institution.

Alcohol intake was prevalent in 21.0% of the subjects which was nearly similar to the studies by Rustagi N et al. (28.8%) and Nanjesh SK et al. (27.0%) [10, 30]. Paul B et al. reported significantly lower values (5.0%) which can be due to social norms prevalent in that part of the country [9].

Intake of tobacco and alcohol were significantly more prevalent among men similar to other Indian data [10]. But contrary to the study from coastal city of Karnataka, tobacco and alcohol use was more prevalent among junior students in this study, which can be due to the lack of awareness and more sedentary lifestyle.

### Overweight, obesity and increased Waist-to-height ratio

Most of the students were overweight or obese in this study (70%) with the proportion being more in junior students (78.1%) which must be due to the sedentary habits, altered food habits in hostel, alcohol consumption and the covid pandemic induced alteration in lifestyle. Overweight and obesity were prevalent in medical students in other studies across the world as well (25–38%) but such high proportions are only reported by the studies by Mahmoud AE et al. and Ofori EK et al. where obesity was prevalent in 61.6% of the Saudi Arabian students and overweight or obesity were prevalent in 53.4% of students from Ghana respectively [9–12, 31, 35, 37].

Increased waist circumference was noted in 26.1% of the subjects, more prevalent in juniors (29.6%) which was higher than 23.0% reported from central Kerala and 15.2% from coastal city of Karnataka, reason probably being same as discussed above [10, 31].

Unlike the other studies among medical students from India or abroad, this study analysed the Waist-to-height ratio and found it to correlate significantly with cardiovascular disease risk ratio, the strength of correlation increasing slightly when applied among non-overweight and non-obese subjects. As pointed out by Ashwell et al., high Waist-to-height ratio in subjects with “healthy” body mass index can be an indicator of ‘early health risk [22]. Hence the use of Waist-to-height ratio even in medical students with normal body mass index can be an early marker of high cardiovascular disease risk.

While overweight and obesity were slightly more prevalent among men, increased waist circumference was significantly more prevalent among women in this study.

### Blood pressure

High systolic blood pressure and diastolic blood pressure were noted in 18.5% and 13.9% subjects respectively. While high systolic blood pressure had nonsignificant gender-wise or seniority wise difference in prevalence, high diastolic blood pressure was significantly more common among men and juniors (66.3% of the juniors were men). This difference can be attributed to more smoking and alcohol consumption in men. The prevalence was similar to those found in studies by Paul B et al. and Kurian S et al. whereas, Nanjesh SK et al. and Thomas A et al. found negligible proportion subjects with high blood pressure [8–10, 31]. Ibrahim NK et al. found high systolic and high diastolic blood pressures in 3.7% and 7.9% of the Saudi Arabian students, on the other hand Ofori EK et al. noted high systolic and high diastolic blood pressures in 45% and 32.5% of the students from Ghana [11]. These wide variations can be attributed to the socio-cultural and genetic differences across the different populations and needs to be taken into consideration while planning preventive and curative strategies.

#### Lipid profile

High low-density lipoprotein was observed in 29.3% subjects, high triglyceride in 33.9%, High total cholesterol in 19.2% which were also more prevalent in men and among junior students may be because bulk of junior students included in the study were men as discussed earlier. Low high-density lipoprotein was prevalent in 17.8% with female predominance. Hypercholesterolemia was present in 17.2% of the students in King Abdulaziz University, Jeddah, Saudi Arabia while dyslipidemia was reported to be present in 6.1% in another study on Egyptian and Saudi Arabian medical students [11, 37]. Triglycerides, total cholesterol and low-density lipoprotein were high in 4.2%, 30% and 67.5% of the nursing students from Ghana while high-density lipoprotein was low in 32.5% [12]. The study by Kurian S et al. found the prevalence of high low-density lipoprotein, triglyceride and total cholesterol to be 5.0%, 13.0% and 8% while prevalence of low high-density lipoprotein was more than this study (33.0%) [31]. The difference of low high density lipoprotein may be due to genetic difference, while the lower low density lipoprotein, triglyceride, total cholesterol can be attributed to lesser prevalence of family history of cardiovascular disease, no intake of tobacco or alcohol and better physical activity profile in the study populations. Covid pandemic induced lifestyle changes may have also attributed to the increased prevalence of higher lipid parameters in the current study.

Prevalence of high cardiovascular disease risk ratio did not differ between the two studies probably because of the higher prevalence of low high-density lipoprotein in the study from central Kerala [30]. Prevalence of high cardiovascular disease risk ratio was also similar to the study from Ghana [12].

### Comparison with general population

In the current study prevalence of high systolic blood pressure and diastolic blood pressure were noted in 18.5% and 13.9% subjects respectively with 5.5% subjects being known hypertensives. 13.4% subjects had high FPG, 4.4% were known type 2 diabetics and 1 was type 1 diabetic. 29.3% were tobacco users. High low-density lipoprotein, triglyceride, total cholesterol and low high density lipoprotein were noted in 29.3%, 33.9%, 19.2% and 17.8% respectively and 12.9% had known dyslipidemia. Large prevalence studies from Indian adults found hypertension to be prevalent in 10.6–39.0%, diabetes to be prevalent in 1.5%- 21.0%, high cholesterol to be present in 23.0–54.1%, smoking in 18.1–42.0% [38].

### Correlation between risk factors

In this study, correlation was noted between body mass index, waist to height ratio, triglyceride, low density lipoprotein and cardiovascular disease risk ratio. Similarly, Ofori EK et al. found strong correlations between low density lipoprotein and CVD risk ratio and moderate correlation between total cholesterol and cardiovascular disease risk ratio [12].

## Limitations

Modification of QRISK2 including smokeless tobacco has been proven to be an important tool for cardiovascular disease risk stratification among Indians but the age group in QRISK 2 and QRISK 3 is 25–84 years [39, 40]. The age group for another important tool, Framinham Risk Score is 30–79 years while for Pooled cohort equation is 40- 79 years [41, 42]. Berry et al. demonstrated that Framinham Risk score and ATP III risk score failed to work in individuals younger than 30 years [42]. Thus total cholesterol/high density lipoprotein ratio was chosen as cardiovascular disease risk ratio as it has been proven to be useful as cardiovascular disease risk estimator and it was easy to use [25]. But multifactorial assessment of cardiovascular disease risk has been compromised due to the inability to use the newer risk scores.

Another major limitation of the study was the comparison between two generation of students. Evaluation of the same population from junior to senior levels would have given clearer conclusion about the success of medical education and development of awareness. Multi-centre based prospective study including larger sample size comprising of undergraduates, interns, postgraduates would give a clearer idea regarding the cardiovascular disease risk factors among medical students in Indian subcontinent. One has to keep in mind that medical undergraduates may not represent the general youth. Covid-19 pandemic scenario may have affected the physical activity and mental stress of the students. Food habits, Lipoprotein (a), High sensitivity C-reactive protein, homocysteine, hemoglobin A1C, uric acid was not included in analysis.

## Conclusions

High prevalence of different modifiable cardiovascular disease risk factors revealed among the subjects in this study is concerning and needs implementation of screening programs, awareness initiatives and surveillance activities. Medical core curriculum should be modified in the direction of awareness and prevention. Regulatory and legislative actions for in-campus tobacco and alcohol use are required. Further research is warranted for the assessment of waist to height ratio (WHtR) as an independent early predictor of cardiovascular disease risk in Indian population and development of a cardiovascular disease risk assessment tool for the young population, given the steep rise of cardiovascular disease in young population across the globe.

## Supplementary Information

**Additional file 1: Table S1**. Gender-wise comparison of cardiovascular risk factors prevalence among students. **Table S2.** Comparison of cardiovascular risk factors prevalence among junior students and senior students. **Table S3.** Association of the baseline parameters with CVD risk ratio

## Data Availability

Available when requested.

## Abbreviations

BMI: Body mass index
DBP: Diastolic blood pressure
FPG: Fasting plasma glucose
HDL: High-density lipoprotein
LDL: Low-density lipoprotein
SBP: Systolic blood pressure
TC: Total cholesterol
TG: Triglyceride
WHtR: Waist-to-height ratio
CVD: Cardiovascular disease
F/H: Family history
PSS: Perceived stress scale
