# Supplementary material for: Cardiovascular disease risk factors among undergraduate medical students in a tertiary care centre of eastern India: a pilot study
Source: Egypt Heart J. 2021 Oct 26;73:94. doi: 10.1186/s43044-021-00219-9 (PMC8547573; doi:10.1186/s43044-021-00219-9)
Supplement: Supplementary file 1 — Additional file 1: Table S1. Gender-wise comparison of cardiovascular risk factors prevalence among students. Table S2. Comparison of cardiovascular risk factors prevalence among junior students and senior students. Table S3. Association of the baseline parameters with CVD risk ratio [file 43044_2021_219_MOESM1_ESM.docx]

**Table S1: Gender-wise comparison of cardiovascular risk factors prevalence among students**

| Variable | Male (n= 263) | Female (n= 170) | Total (n= 433) | p-value |
| --- | --- | --- | --- | --- |
| PSS low | 64 (24.3) | 52 (30.6) | 116 (26.8) | 0.03^*^ |
| PSS average | 131(49.8) | 89 (52.4) | 220 (50.8) |  |
| PSS high | 68 (25.9) | 29 (17.1) | 97 (22.4) |  |
| Physical activity- low | 76 (28.9) | 52 (30.6) | 128 (29.6) | 0.7 |
| Physical activity- moderate | 187 (71.1) | 118 (69.4) | 305 (70.4) |  |
| F/H of CVD | 61 (23.2) | 30 (17.6) | 91 (21.0) | 0.2 |
| F/H of DM | 77 (29.3) | 53 (31.2) | 130 (30.0) | 0.7 |
| F/H of Dyslipidemia | 65 (24.7) | 28 (16.5) | 93 (21.5) | 0.04^*^ |
| Tobacco intake | 103 (39.2) | 24 (14.1) | 127 (29.3) | <0.001^*^ |
| Alcohol intake | 83 (31.6) | 8 (4.7) | 91 (21.0) | <0.001^*^ |
| Underweight | 4 (1.5) | 0 (0.0) | 4 (0.9) | 0.7 |
| Normal | 71 (27.0) | 55 (32.4) | 126 (29.1) |  |
| Overweight | 46 (17.5) | 25 (14.7) | 71 (16.4) |  |
| Obese | 142 (54.0) | 90 (52.9) | 232 (53.6) |  |
|  |  |  |  |  |
| SBP <120 mm Hg | 40 (15.2) | 43 (25.3) | 83 (19.2) | 0.3 |
| SBP 120-129 mm Hg | 117 (44.5) | 54 (31.8) | 171 (39.5) |  |
| SBP 130-139 mm Hg | 54 (20.5) | 45 (26.5) | 99 (22.9) |  |
| SBP ≥140 mm Hg | 52 (19.8) | 28 (16.5) | 80 (18.5) |  |
| DBP <80 mm Hg | 51 (19.4) | 88 (51.8) | 139 (32.1) | <0.001^*^ |
| DBP 80-89 mm Hg | 168 (63.9) | 66 (38.8) | 234 (54.0) |  |
| DBP ≥ 90 mm Hg | 44 (16.7) | 16 (9.4) | 60 (13.9) |  |
| Hypertension | 60 (22.8) | 32 (18.8) | 92 (21.2) | 0.3 |
| Diabetes | 44 (16.7) | 14 (8.2) | 58 (13.4) | 0.01^*^ |
| Increased Waist circumference | 12 (4.6) | 101 (59.4) | 113 (26.1) | <0.001^*^ |
| High WHtR | 114 (43.3) | 90 (52.9) | 204 (47.1) | 0.051 |
| High LDL | 93 (35.4) | 34 (20.0) | 127 (29.3) | 0.001^*^ |
| High TG | 103 (39.2) | 44 (25.9) | 147 (33.9) | 0.004^*^ |
| High TC | 66 (25.1) | 17 (10.0) | 83 (19.2) | <0.001^*^ |
| Low HDL | 26 (9.9) | 51 (30.0) | 77 (17.8) | <0.001^*^ |
| High CVD risk ratio | 50 (19) | 12 (7.1) | 62 (14.3) | <0.001^*^ |

*test significant at *p* < 0.05. Discrete variables are expressed as n (%).

CVD- cardiovascular disease; DBP- diastolic blood pressure; DM- diabetes; F/H- family history; HDL- high density lipoprotein; LDL- low density lipoprotein; PSS- perceived stress scale; SBP- systolic blood pressure; TC- total cholesterol; TG- triglyceride

**Table S2: Comparison of cardiovascular risk factors prevalence among junior students and senior students**

| Variable | Junior students (n= 297) | Senior students (n= 136) | Total (n= 433) | Significance |
| --- | --- | --- | --- | --- |
| PSS low | 84 (28.3) | 32 (23.5) | 116 (26.8) | <0.001^*^ |
| PSS average | 132 (44.4) | 88 (64.7) | 220 (50.8) |  |
| PSS high | 81 (27.3) | 16 (11.8) | 97 (22.4) |  |
| Physical activity- low | 108 (36.4) | 20 (14.7) | 128 (29.6) | <0.001^*^ |
| Physical activity- moderate | 189 (63.6) | 116 (85.3) | 305 (70.4) |  |
| F/H of CVD | 61 (20.5) | 30 (22.1) | 91 (21.0) | 0.7 |
| F/H of DM | 100 (33.7) | 30 (22.1) | 130 (30.0) | 0.01^*^ |
| F/H of Dyslipidemia | 83 (27.9) | 10 (7.4) | 93 (21.5) | <0.001^*^ |
| Tobacco intake | 105 (35.4) | 22 (16.2) | 127 (29.3) | <0.001^*^ |
| Alcohol intake | 74 (24.9) | 17 (12.5) | 91 (21.0) | 0.003 |
| Underweight | 4 (1.3) | 0 (0.0) | 4 (0.9) | <0.001^*^ |
| Normal | 61 (20.5) | 65 (47.8) | 126 (29.1) |  |
| Overweight | 62 (20.9) | 9 (6.6) | 71 (16.4) |  |
| Obese | 170 (57.2) | 62 (45.6) | 232 (53.6) |  |
| SBP <120 mm Hg | 39 (13.1) | 44 (32.4) | 83 (19.2) | <0.001^*^ |
| SBP 120-129 mm Hg | 126 (42.4) | 45 (33.1) | 171 (39.5) |  |
| SBP 130-139 mm Hg | 76 (25.6) | 23 (16.9) | 99 (22.9) |  |
| SBP ≥140 mm Hg | 56 (18.9) | 24 (17.6) | 80 (18.5) |  |
| DBP <80 mm Hg | 73 (24.6) | 66 (48.5) | 139 (32.1) | <0.001^*^ |
| DBP 80-89 mm Hg | 172 (57.9) | 62 (45.6) | 234 (54.0) |  |
| DBP ≥ 90 mm Hg | 52 (17.5) | 8 (5.9) | 60 (13.9) |  |
| Hypertension | 68 (22.9) | 24 (17.6) | 92 (21.2) | 0.2 |
| Diabetes | 29 (9.8) | 29 (21.3) | 58 (13.4) | 0.001^*^ |
| Increased Waist circumference | 88 (29.6) | 25 (18.4) | 113 (26.1) | 0.01^*^ |
| High WHtR | 157 (52.9) | 47 (34.6) | 204 (47.1) | <0.001^*^ |
| High LDL | 98 (33.0) | 29 (21.3) | 127 (29.3) | 0.01^*^ |
| High TG | 113 (38.0) | 34 (25.0) | 147 (33.9) | 0.008^*^ |
| High TC | 67 (22.6) | 16 (11.8) | 83 (19.2) | 0.008^*^ |
| Low HDL | 57 (19.2) | 20 (14.7) | 77 (17.8) | 0.3 |
| High CVD risk ratio | 46 (15.5) | 16 (11.8) | 62 (14.3) | 0.3 |

*test significant at *p* < 0.05. Discrete variables are expressed as n (%).

CVD- cardiovascular disease; DBP- diastolic blood pressure; DM- diabetes; F/H- family history; HDL- high density lipoprotein; LDL- low density lipoprotein; PSS- perceived stress scale; SBP- systolic blood pressure; TC- total cholesterol; TG- triglyceride

**Table S3: Association of the baseline parameters with CVD risk ratio**

| Variable | TC/HDL ratio high (n= 62) | TC/HDL ratio acceptable (n= 371) | OR (95% CI) | Chi-square (LR) | p-value |
| --- | --- | --- | --- | --- | --- |
| Women | 12 (19.4) | 158 (42.6) | 0.32 (0.17- 0.63) | 12.02 (13.08) | 0.001 |
| Junior students | 46 (74.2) | 251 (67.7) | 0.73 (0.39- 1.34) | 1.05 (1.08) | 0.3 |
| Rural residence | 24 (38.7) | 140 (37.7) | 1.04 (0.60- 1.81) | 0.02 (0.02) | 0.9 |
| High PSS | 20 (32.3) | 77 (20.8) | 0.55 (0.31- 0.99) | 4.04 (3.76) | 0.04 |
| Low physical activity | 32 (51.6%) | 96 (25.9%) | 0.33 (0.19- 0.57) | 16.89 | <0.001 |
| F/H of CVD | 69 (18.6) | 22 (35.5) | 0.42 (0.23- 0.74) | 9.13 (8.21) | 0.003 |
| Tobacco intake | 30 (48.4) | 97 (26.1) | 0.38 (0.22- 0.65) | 12.68 (11.78) | <0.001 |
| Overweight and obese | 54 (87.1) | 249 (67.1) | 0.30 (0.14- 0.66) | 10.09 (11.55) | 0.001 |
| Hypertension | 20 (32.3) | 72 (19.4) | 0.51 (0.28- 0.91) | 5.24 (4.82) | 0.02 |
| Diabetes | 14 (22.6) | 44 (12.0) | 0.46 (0.24- 0.91) | 5.26 (4.64) | 0.02 |
| High WHR (>0.5) | 42 (67.7) | 162 (43.8) | 0.37 (0.21- 0.65) | 12.23 (12.38) | <0.001 |
| High LDL (> 100 mg/dL) | 58 (93.5) | 69 (18.6) | 63.46 (22.29- 180.71) | 143.97 (137.9) | <0.001 |
| High TG (>150 mg/dL) | 36 (58.1) | 111 (29.9) | 3.24 (1.87- 5.63) | 18.77 (17.76) | <0.001 |

CI- Confidence interval; CVD- cardiovascular disease; F/H- family history; LDL- low density lipoprotein; LR- likelihood ratio; OR- Odds ratio; PSS- perceived stress scale; TG- triglyceride; WHtR- waist-to-height ratio
